# p53 gene analysis in childhood B non -Hodgkin's lymphoma

**DOI:** 10.1590/S1516-31802001000600006

**Published:** 2001-11-01

**Authors:** Claudete Esteves Nogueira Pinto Klumb, Lídia Maria Magalhães de Resende, Eloísa Helena Tajara, Erika Cristina Pavarino Bertelli, Vivian Mary Rumjanek, Raquel Ciuvalschi Maia

**Keywords:** p53 mutation, B non-Hodgkin's lymphoma, Burkitt's lymphoma, Mutação do gene p53, Linfoma não-Hodgkin, Linfoma de Burkitt

## Abstract

**CONTEXT:**

Mutations or deletions in the tumor-suppressor gene p53 are among the commonest genetic changes found in human neoplasms including breast, lung and bowel cancers. In hematological malignancies, p53 is most often mutated in Burkitt's lymphoma, with p53 mutations present in 30 to 40% of tumor samples and in 70% of cell lines.

**OBJECTIVE:**

To analyze the p53 gene alterations in child patients with B non-Hodgkin's lymphoma.

**DESIGN:**

Descriptive study.

**SETTING:**

Tertiary oncology care center.

**PARTICIPANTS:**

The study investigated 12 patients with childhood B non-Hodgkin's lymphoma (Burkitt's lymphoma). Screening for p53 mutations was done by polymerase chain reaction - single strand conformational polymorphism(PCR-SSCP)analysis of exon 5 to 8/9 of the gene.

**RESULTS:**

Abnormal polymerase chain reaction - single strand conformational polymorphism migration pattern was observed in 4 patients (33.3%), one on exon 6 and three on exon 7. Positive cases included 2 patients who died from disease.

**CONCLUSION:**

These preliminary results suggest that p53 mutations are quite frequent in children with Burkitt's lymphoma and may play a role in lymphoma genesis or disease progression.

## RESUMO

**CONTEXTO:**

Alterações do gene supressor de tumor p53, como mutações e deleções, são lesões genéticas encontradas com maior freqüência nas neoplasias humanas, incluindo câncer de mama, pulmão e cólon. Entre as malignidades hematológicas, o gene 53 é freqüentemente mutado no linfoma de Burkitt, sendo detectadas mutações em 3040% das amostras tumorais e em 70% das linhagens celulares.

**OBJETIVO:**

Analisar as alterações do gene p53 em crianças com linfoma não-Hodgkin de origem B.

**TIPO DE ESTUDO:**

Estudo descritivo. **LOCAL:** Centro de Oncologia Terciário. **PARTICIPANTES:** O estudo analisou 12 pacientes com linfoma não-Hodgkin B classificados como linfoma de Burkitt. A análise de possíveis mutações do gene p53 foi realizada pela técnica de PCR-SSCP dos exons 5, 6, 7 e 8/9 do gene.

**RESULTADOS:**

Um padrão anormal de migração foi observado em quatro pacientes (33.3%), em um paciente no exon 6 e em três no exon 7. Os casos positivos incluíam dois pacientes que evoluíram para o óbito por progressão da doença.

**CONCLUSÃO:**

Esses resultados preliminares sugerem que as alterações do gene p53 são freqüentes em crianças com linfoma de Burkitt e podem contribuir para patogênese ou progressão da doença.

## INTRODUCTION

p53 is a tumor-suppressor gene whose disruption or loss is implicated in development or progression of several types of human cancer.^1-3^ Its inactivation is mainly caused by point mutations in the coding sequence of exons 5 to 9 in one allele, with or without loss of the other allele.^4^ Burkitt's lymphoma is a subgroup of nonHodgkin's lymphoma (NHL) with distinct epidemiological, clinical-pathological, immunological, and molecular features. It occurs as an Epstein-Barr virus associated with B nonHodgkin's lymphoma with high incidence among children, in the malaria belt of equatorial Africa (endemic Burkitt's lymphoma) and sporadically in other geographical areas, when it is named sporadic Burkitt's lymphoma.^5^ The characteristic genetic marker of Burkitt's lymphoma is a reciprocal translocation involving the c-myc gene on chromosome 8 and one of three immunoglobulin gene loci, most commonly the heavy chain gene locus on chromosome 14 and less frequently the light chain gene loci on chromosome 2 and 22.^6^ Evidence from several studies has led to the conclusion that c-myc translocation is not the only event in the pathogenesis of Burkitt's lymphoma. Occurrence of additional pathogenic steps like tumor cell infection with Epstein-Barr virus and mutations in the regulatory and coding regions of c-myc might contribute to the oncogenic process.^7^^,^^8^

Experimental studies suggest that alterations in the p53 gene might be relevant to the development of lymphomas in transgenic mice carrying mutant p53 alleles.^9^ A very strong correlation between p53 status and tumor-proneness among nude mice has been observed for Burkitt's lymphoma cell lines but not for other cell types.^10^

p53 protein is necessary to induce the response to many chemotherapeutic drugs used for treatment of Burkitt's lymphoma. The present chemotherapeutic protocols are highly effective, although they are not free from adverse effects. Therefore, in the context of resistance/sensitivity to treatment, absence of p53 mutation could be an important finding for selecting patients that could be treated with a less intensive chemotherapeutic schedule.

In order to establish the frequency of p53 alterations among B non-Hodgkin's lymphoma in children, especially in Burkitt's lymphoma, we studied 12 newly diagnosed patients.

## METHODS

### Samples

Twelve cases of untreated B non-Hodgkin's lymphoma (all of which were Burkitt's lymphoma) were evaluated. The cases were classified according to the non-Hodgkin's Lymphoma Pathologic Classification Project.^11^ B-cell phenotype was confirmed by immunostaining with CD20 monoclonal antibody L26.

DNA was obtained from paraffin-embedded tumor tissue and extracted using previously published methods.^12^^,^^13^

### Polymerase chain reaction - single strand conformational polymorphism (PCR-SSCP) of the p53 gene

Single strand conformational polymorphism analysis was based on the method reported by Orita et al.^14^ Polymerase chain reaction was performed with 100-500 ng of genomic DNA, 25 pmol of each primer exon 5, 6, 7 and 8/9, 0.2 mM dNTP, 1 U Taq polymerase (GIBCO-BRL) in a final volume of 45 mL (exon 6, 25 mL). Thirty-four cycles of denaturation (94 °C), annealing (annealing temperatures were optimized for each pair of primers), and extension (72 °C) were performed in a thermocycler (Minicycler^TM^, MJ Research). The four exons were amplified separately. After amplification, 5mL of reaction mixture was mixed with 10 mL 98% formamide / 10 mM EDTA, 0.025% bromophenol blue, and 0.025% xylene cyanol. Samples were heated at 95 °C for 5 min, chilled on ice and immediately loaded on a 7.5% polyacrylamide - TBE gel. We performed the electrophoresis at room temperature, with or without the addition of glycerol, or at 4 °C in a cold room. Gels were run at 3 W for 4 h, stained with silver nitrate and air-dried.

## RESULTS

Clinic pathological data of patients are listed in the Table. The patients ranged in age from 4 to 9 years (median = 5.5 years). Male/female ratio was 2:1. According to the St. Jude staging system,^15^ 16.6%% of patients had stage I/II disease, 66.6% stage III and 16.6% stage IV disease. All patients had a histological diagnosis corresponding to Burkitt's lymphoma.

**Table:** Clinical pathological data and outcome for patients with Burkitt's lymphoma

*Patient*	*Sex*	*Age (years)*	*Primary Site of tumor*	*Disease Stage*	*HIV*	*Survival (months)*	*DFS (months)*	*Outcome*
								
***1***	***M***	***5***	***Cervical node***	***I***	***Neg***	***28***	***26***	***Alive***
***2***	***M***	***6***	***Nasopharynx***	***I***	***Neg***	***4***	***0***	Dead*
***3***	***F***	***4***	***Abdomen***	***III***	***Neg***	***7***	***2***	Dead*
***4***	***M***	***5***	***Abdomen***	***III***	***Neg***	***5***	***0***	***Dead¶***
***5***	***M***	***8***	***Abdomen***	***III***	***Neg***	***21***	***19***	***Alive***
***6***	***M***	***4***	***Abdomen***	***III***	***Neg***	***21***	***18***	***Alive***
***7***	***F***	***4***	***Abdomen/pelvis***	***II***	***Neg***	***38***	***36***	***Alive***
***8***	***M***	***7***	***Abdomen***	***III***	***Neg***	***4***	***0***	Dead*
***9***	***M***	***6***	***Abdomen***	***III***	***Neg***	***54***	***49***	***Alive***
***10***	***M***	***5***	***Abdomen***	***III***	***ND***	***84***	***82***	***Alive***
***11***	***F***	***9***	***Abdomen/BM***	***IV***	***Neg***	***64***	***62***	***Alive***
***12***	***F***	***6***	***Pelvis/CNS***	***IV***	***Neg***	***21***	***19***	***Alive***

***Abbreviations: M, male;F, female;BL, Burkitt's lymphoma;BM, bone marrow;DFS, disease free survival;Neg, negative;ND, not determined.***

* Death related to refractory disease;¶Death(quit treatment).

The outcome for all patients is shown in the Table. Eleven patients achieved complete remission, 8/12 remain alive. Four patients died because of failure to achieve complete remission, relapse or non-adherence to treatment.

Upon polymerase chain reaction-single strand conformational polymorphism analysis, four p53 gene alterations were demonstrated. These four alterations consisted of one on exon 6 and three on exon 7.

The figure shows the results of polymerase chain reaction-single strand conformational polymorphism analysis for exons 6 and 7. The polymerase chain reaction products of exon 6 (lane 5) and exon 7 (lanes 2, 4, 9) showed mobility shifts upon single strand conformational polymorphism analysis. Positive cases included 2 patients who died from disease.

**Figure:**
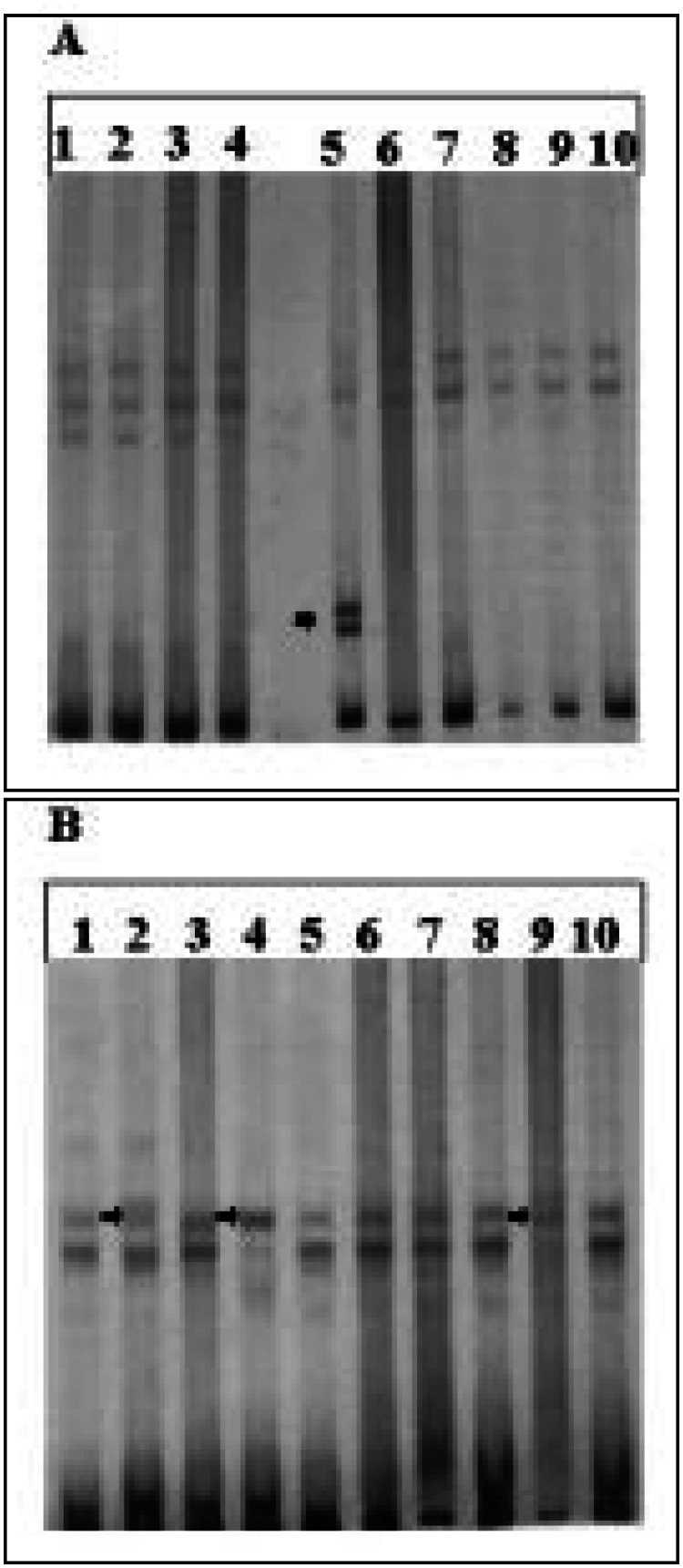
Polymerase chain reaction-single strand conformotional polymorphism analysis of p53 gene. Amplified genomic DNA fragments corresponding to exons 5-9 were denatured by heating and electrophoresis was performed in 7.5% polyacrylamide gel. DNA samples suggestive of having p53 mutations show shifts in electrophoretic mobility compared to other DNA samples on the same gel. Arrows show shifts in electrophoretic mobility **(A)**: Exon 6. Lane 5 corresponds to patient 1 **(B)**: Exon 7. Lanes 2,4,9 correspond to patients 3,5 and 8, respectively.

The polymerase chain reaction products of exon 5 and exons 8/9 showed no mobility shifts. To confirm that our experiments were free from contamination or some other artifacts, all experiments were repeated using another section cut from the same block of paraffin embedded tissue.

## DISCUSSION

We examined 12 cases of childhood B nonHodgkin's lymphoma for alterations of p53 gene using polymerase chain reaction-single strand conformational polymorphism analysis, which is a simple detection system for point mutations.^16,17^ An abnormal single strand conformational polymorphism migration pattern was detected in four samples (33.3%). Polymerase chain reaction-single strand conformational polymorphism analysis is a technique based on the three-dimensional conformation taken by a single-stranded DNA in a non-denaturing environment where any change in the sequence can result in a variation of the electrophoretic mobility. The specificity of polymerase chain reaction-single strand conformational polymorphism is more than 95% for 100 to 300 bp polymerase chain reaction fragments.^17^ In practice, not all of the changes can be resolved but the modification of the migration conditions can improve the detectability of the mutations along the sequence. The polymerase chain reaction-single strand conformational polymorphism protocol is a very useful screening method for detecting mutations in a short region of a gene. However, the samples with abnormal single strand conformational polymorphism require nucleotide sequencing studies to assert whether the abnormal migration represents a mutation or a polymorphism.

Several groups have found that p53 is usually mutated in Burkitt's lymphoma cell lines.^18^^-^^20^ The frequency of p53 mutations in fresh tumor samples has previously been reported as 40%. ^18,21^ We found that 33.3% of paraffin embedded samples from children studied at diagnosis of Burkitt's lymphoma had p53 alterations suggestive of gene mutation. Our results were similar to those reported by other authors,^18,21^ who found 33% to 37%. However, a recent study has shown 19% of p53 mutations in newly diagnosed adult and child patients with Burkitt's lymphoma.^22^

In our study, all the Burkitt's patients were children studied at diagnosis. In the study by Preudhomme et al.,^22^ there were only 2/12 children with p53 alterations among the patients studied (16%). Bathia et al.^21^ found 37% of p53 mutations in patients with Burkitt's lymphoma from Argentina and Brazil, although there was no reference to how many children were included and whether they represented newly diagnoses or relapse of disease. Higher percentages of p53 mutations would be expected in relapse situations.

In a previous report studying a large series of childhood lymphoid malignancies, p53 mutations were detected in 2/8 B non-Hodgkin's lymphoma.^23^ The total number of cases examined by that group was too small to determine the frequency of p53 mutation in B non-Hodgkin's. Among all the types of lymphoid neoplasm, the most frequent targets for p53 mutations are Burkitt's lymphoma and its leukemic counterpart L_3_-type B-cell acute lymphoblastic leukemia.^18^

The use of p53 gene mutations as a potential prognostic marker in lymphoid childhood malignancies is limited by its low frequency at diagnosis. Nevertheless, the higher frequency of p53 in Burkitt's lymphoma patients observed by Gaidano et al^18^ and others^21^ suggests that p53 mutations can be found in children with Burkitt's lymphoma.

In our study, four Burkitt's patients with abnormal p53 migration were found. Of those, two died as a result of lack of response to treatment and consequent disease progression, but two are still alive.

In a large panel of Burkitt's lymphoma cell lines,^18,24^the normal allele had been lost in a large number of lines or both alleles present had undergone point mutations. Burkitt's lymphoma cell lines carrying a p53 mutation are more radioresistant when they lose the normal p53 allele than when they retain it.^25^ Preudhomme et al.^22^ found persistence of the normal p53 allele in most newly diagnosed Burkitt's lymphoma patients with mutations. The loss of the normal allele in cell lines suggests that this is a late event in the evolution of Burkitt's lymphoma, especially in relapsed patients and may be responsible for the resistance to chemotherapy observed in those tumors upon relapse. Hence, particular forms of mutant p53 may directly enhance the resistance of tumor cells to anticancer agents and confer a selective survival advantage during chemotherapy.^26^The relevance of these findings should be extended to cells lines and patients with Burkitt's lymphoma.

These observations suggest that p53 gene alteration in tumors carrying c-myc protooncogene activation in children can be detected in newly diagnosed patients and play a role in lymphoma genesis or disease progression.

Burkitt's lymphoma includes two pathogenically distinct forms, namely the endemic African type characterized by Epstein-Barr virus infection and the sporadic American type characterized by Epstein-Barr virus infection in only 30% of cases.^27^ Higher frequencies of Epstein-Barr virus infection (71 to 87%) have been found in Brazil.^28^^,^^29^

A common denominator among all these diseases is the consistent presence of chromosomal translocation leading to c-myc activation.^6^ On the other hand, environmental factors or lifestyle acts on the molecular subtypes of Burkitt's lymphoma.^30^ It may possible that ethnic and/or geographical factors might account for different frequencies of p53 alterations between Brazilian Burkitt's lymphoma and sporadic Burkitt's lymphoma patients from others countries.
